# Genome-Wide Profiling of DNA Methylome and Transcriptome Reveals Epigenetic Regulation of Potato Response to DON Stress

**DOI:** 10.3389/fpls.2022.934379

**Published:** 2022-06-23

**Authors:** Yan Shi, Yuan Qin, Fenglan Li, Haifeng Wang

**Affiliations:** ^1^College of Plant Protection, Fujian Agriculture and Forestry University, Fuzhou, China; ^2^College of Life Science, Northeast Agricultural University, Harbin, China; ^3^State Key Laboratory for Conservation and Utilization of Subtropical Agro-Bioresources, Guangxi Key Lab for Sugarcane Biology, College of Agriculture, Guangxi University, Nanning, China

**Keywords:** DNA methylation, transcriptome, potato, dry rot disease, deoxynivalenol

## Abstract

Potato is an important food crop that occupies lesser area but has greater production than rice and wheat. However, potato production is affected by numerous biotic and abiotic stresses, among which *Fusarium* dry rot is a disease that has significant effect on potato production, storage, and processing. However, the role of DNA methylation in regulating potato response to *Fusarium* toxin deoxynivalenol (DON) stress is still not fully understood. In this study, we performed DNA methylome and transcriptome analyses of potato tubers treated with five concentrations of DON. The global DNA methylation levels in potato tubers treated with different concentrations of DON showed significant changes relative to those in the control. In particular, the 20 ng/ml treatment showed the largest decrease in all three contexts of methylation levels, especially CHH contexts in transposon regions. The differentially methylated region (DMR)-associated differentially expressed genes (DEGs) were significantly enriched in resistance-related metabolic pathways, indicating that DNA methylation plays an essential role in potato response to DON stress. Furthermore, we examined lesions on potato tubers infested with *Fusarium* after treatment. Furthermore, the potato tubers treated with 5 and 35 ng/ml DON had lesions of significantly smaller diameters than those of the control, indicating that DON stress may induce resistance. We speculate that this may be related to epigenetic memory created after DNA methylation changes. The detailed DNA methylome and transcriptome profiles suggest that DNA methylation plays a vital role in potato disease resistance and has great potential for enhancing potato dry rot resistance.

## Introduction

Potato (*Solanum tuberosum* L.) is an important non-cereal food crop that has been cultivated for more than 8,000 years and has great potential to ensure food security in developing countries. It has an annual global production of 388 million tons cultivated in an area of 19 million hectares (FAOSTAT, 2019). Recent study showed that the potato genome consists of 66% repetitive sequences, of which long terminal repeat retrotransposons are the most abundant class (Pham et al., 2020). The water content of potato tubers exceeds 70%, rendering them vulnerable to rotting during handling, transportation, and storage after harvest; rot severely affects the quality of tuber flesh. There are two major types of rot: soft rot and dry rot. Potato dry rot, a devastating fungal disease caused by *Fusarium* species, can cause losses of up to 25% with infections as high as 60% during storage (Fiers et al., 2012; Bojanowski et al., 2013). In addition to being pathogenic, *Fusarium* can produce mycotoxins in plant species (Bojanowski et al., 2013; Perincherry et al., 2019). Some of these mycotoxins also act as pathogenic factors in the development of rot in tubers while affecting food safety. Therefore, dry rot is a threat to potato production and processing.

Treatment of plants with plant pathogens or elicitors can activate basal defense systems and generate induced resistance to pathogens as well as to future pathogen challenges (Reimer-Michalski and Conrath, 2016; Mauch-Mani et al., 2017). This induced resistance can be maintained over generations (Slaughter et al., 2012; Iwasaki and Paszkowski, 2014). For example, bacterial species such as *Bacillus* spp. and *Pseudomonas* spp. can stimulate defense responses and help plants acquire disease resistance (Van Peer et al., 1991; Ongena et al., 2005). The priming agent β-amino-butyric acid (BABA) treatment of common bean (*Phaseolus vulgaris* L.) also enhanced plant resistance against bacterial pathogens (*Pseudomonas syringae* pv. Phaseolicola; Ramírez-Carrasco et al., 2017). Treatment of *Arabidopsis thaliana* with low concentrations of T-2, a type-A monoterpene produced by *Fusarium*, induced resistance to *Fusarium* (Nishiuchi et al., 2006). Notably, low concentrations of T-2 treated potato tubers induced an increased abundance of reactive oxygen species (ROS) and new phenylpropanoid metabolites, enhancing resistance against dry rot (Xue et al., 2019). Epigenetic analyses have demonstrated that DNA methylation is not only involved and regulated in plant resistance responses but is also associated with the establishment of induced resistance (Lämke and Bäurle, 2017; He and Li, 2018).

DNA methylation is an epigenetic marker conserved in plants and plays a vital role in regulating gene expression, stabilizing the genome, plant development, and environmental adaptation (Mendizabal and Yi, 2016; Kumar et al., 2017, 2018). In plants, DNA methylation can occur in three sequence contexts: CG, CHG, and CHH (H for A, T, or C; Lister et al., 2008). DNA methylation patterns are the result of the coordination of three processes in plants: *de novo* DNA methylation, maintenance, and demethylation. In Arabidopsis, *de novo* DNA methylation involves the RNA-directed DNA methylation (RdDM) pathway, which utilizes small interfering RNAs (siRNAs), scaffold RNAs, and several accessory proteins (Law and Jacobsen, 2010; He et al., 2011; Matzke and Mosher, 2014). Maintenance of DNA methylation in the three cytosine contexts is catalyzed by different enzymes that are regulated by different mechanisms. CG cytosine methylation is performed by METHYLTRANSFERASE (MET1), whereas CHG methylation is mainly catalyzed by the DNA methyltransferase CHROMOMETHYLASE 3 (CMT3) and to a much lesser extent by CMT2 (Lindroth et al., 2001; He et al., 2011; Stroud et al., 2014). Moreover, asymmetric CHH methylation is maintained by CMT2 or DOMAINS REARRANGED METHYLTRANSFERASE 2 (DRM2). DRM2 maintains CHH methylation through the RdDM pathway at shorter transposons and repeat sequences in euchromatic regions (Zemach et al., 2013; Zhang et al., 2018). However, CMT2 mediates CHH methylation of longer TEs in the pericentromeric region (Gouil and Baulcombe, 2016). Additionally, these two enzymes can methylate cytosines in three sequence contexts. DNA demethylases are involved in the base excision repair pathway and initiate active demethylation. In Arabidopsis, the DNA demethylase family comprises four main proteins: REPRESSOR OF SILENCING 1 (ROS1), DEMETER (DME), DEMETER-LIKE PROTEIN 2 (DML2), and DML3 (Gehring et al., 2006; Ortega-Galisteo et al., 2008). DNA methylation is associated with the transcriptional regulation of genes in response to plant stress (Secco et al., 2015; Xu et al., 2015; Satgé et al., 2016; Liu et al., 2017). In general, the methylation status of plant genomic regions is relatively conserved. TEs are usually heavily methylated in three sequence contexts, whereas CG methylation usually occurs in the gene body (Zemach et al., 2010; Mirouze and Vitte, 2014). Increasing evidence suggests that environmental stress can lead to alterations in plant DNA methylation at individual gene loci or across the entire genome. For example, RdDM pathway-mediated DNA methylation dynamically regulates the expression of numerous heat stress-responsive genes in Arabidopsis (Popova et al., 2013). Drought stress induces changes in DNA methylation levels, thereby altering the expression patterns of several drought stress-related genes (Liang et al., 2014). In addition, stress memory in higher plants may be stored in an epigenetic form resulting in greater resilience when encountering sudden environmental changes in the future (Sanchez and Paszkowski, 2014; Wibowo et al., 2016). For instance, treatment of rice with MeJA can alter genome-wide DNA methylation levels, establishing a chromatin-based memory of the stress (Laura et al., 2018).

Deoxynivalenol (DON), a type B trichothecene, is a well-known virulence factor that promotes *Fusarium* spread within wheat spikes (Lemmens et al., 2005) and has also been detected in dry rot of potato tubers (Xue et al., 2013). In a previous study, we found that treating potato tubers with DON also affected resistance to dry rot caused by *Fusarium sambucinum*. However, the effects of mycotoxins on DNA methylation in potato tubers and the relationship between DNA methylation and transcriptional changes have not been described. In this study, we combined single-base resolution bisulfite sequencing (BS-seq) and used to determine the DNA methylation patterns in potato tubers treated with five concentrations of DON, with transcriptome analysis to explore the regulatory effects of DNA methylation in response to DON stress. We found local changes in DNA methylation after treatment with different concentrations of DON through comparative analysis. This study demonstrates the critical role of DNA methylation in the potato response to DON stress and its possible role in the development of induced resistance.

## Materials and Methods

### Plant Materials and Strain

In this study, the plant material Atlantic potato cultivar was provided by the Heilongjiang Provincial Academy of Agricultural Sciences. *F. sambucinum*, the causal strain of dry rot, was stored at the Botany Laboratory of Northeast Agricultural University. *F. sambucinum* was cultured on PDA medium for 15 days at the homothermal condition of 25°C under dark conditions and then inoculated with potatoes. The vomitoxin (DON) was purchased from FERMENTEK.

### Whole-Genome Bisulfite Sequencing and Analysis

DNA was extracted from potato tubers treated with different concentrations of DON using the Qiagen DNeasy Plant Mini Kit and BS-seq libraries were prepared using the TruSeq Nano DNA LT Kit (Illumina). Two biological replicates corresponding to two libraries were constructed for each treatment concentration, and 150-bp paired-end reads were obtained using the HiSeq X Ten System (Illumina) according to the manufacturer’s instructions. Raw data were trimmed using Trimmomatic to retain clean reads. The *S. tuberosum* (v6.1; Pham et al., 2020) genome downloaded from the Phytozome database was used as the reference genome. We used BSMAP (v2.90; Xi and Li, 2009) to map clean data to the reference genome, allowing for four mismatches per 100 bp read length and retaining uniquely mapped reads for further analysis. The methylation information for each cytosine site was extracted after removing the duplicate reads. The methyl-cytosines were evaluated by the binomial test with false discovery rate (FDR) < 0.01. Conversion rates were calculated based on DNA methylation levels from the lamdba genome. Metaplots were generated using the average weighted DNA methylation levels for the gene and TE regions, which contained regions flanking the 2 kb region. DMRs were identified using MethylKit, a comprehensive R package (Akalin et al., 2012). For CG-, CHG-, and CHH-DMRs, the absolute methylation level differences between treatments and controls were required to be at least 0.4, 0.2, and 0.1, respectively. The methylation level of a genomic region was visualized using IGV (v2.11.9).

### RNA Sequencing and Analysis

RNA-seq libraries (three biological replicates per concentration) were constructed using the RNA-seq Library Prep Kit for Illumina. Each library was sequenced using the Illumina HiSeq X Ten System to obtain paired-end 150-bp reads. For RNA-seq data, clean reads were obtained by QC of raw reads using FastQC (v0.11.8) and Trimmomatic (v0.36) and were mapped to the potato reference genome using Hisat2 version 2.2.1.^1^ Expression levels were quantified by Stingtie version 1.3 (Pertea et al., 2016) and were normalized using the TPM (transcripts per kilobase per million mapped reads).^2^ DEGs were identified using DESeq2 (v1.22.2; Love et al., 2014) with default parameters. DEG identification was based on a cutoff value of fold change > 2. GO annotation and enrichment analysis of DMR-associated DEGs using GOATOOLS with a q-value < 0.05 was used to identify the enriched GO terms (Klopfenstein et al., 2018).^3^ Heatmap, and GO enrichment plot of DEGs were made using R software version 3.5.^4^

### *Fusarium* Infestation Experiment

Potato tubers treated with different concentrations of DON were perforated, and *Fusarium* was inoculated in the holes (1 cm diameter). The potato tubers were incubated at 25°C for 10 days and then cut open to determine the size of the spots using the crossover method. The measurements were repeated three times for each group of 30 potatoes.

## Results

### Characteristics of DNA Methylation in Different Concentrations of DON

DNA methylation is widely involved in plant stress responses (Liu and He, 2020). To characterize the DNA methylation patterns in potato tubers treated with DON, genome-wide methylation profiles at single-base resolution were generated using BS-seq for five concentrations of DON (0, 5, 20, 35, and 50 ng/ml), and each treatment was sequenced in two biological replicates. Each library produced at least 180 million reads (read length: 150 bp), forming a total of approximately 300 GB of data (Supplementary Table 1). The reads were mapped to the potato reference genome using BSMAP (Xi and Li, 2009); ~58% of the reads were uniquely mapped to the reference genome, and ~78% of all cytosines in the reference genome were covered by at least four different reads (0 ng/ml as an example, Supplementary Figure 1). The average genome coverage of all sequenced methylation data was ~30-fold, and the average bisulfite conversion rate of the methylated libraries was assessed using lambda DNA sequences, which showed that the average conversion rate of unmethylated cytosine (C) to thymine (T) exceeded 99% (Supplementary Table 1). We assessed the reproducibility of BS-seq data by calculating the Pearson correlation coefficients between two biological replicates of each sample, and the results showed that the Pearson’s correlation coefficients between two biological replicates were ranged from 0.9 to 0.95, indicating a high reproducibility of our methylation data (Supplementary Figure 2). The quality of the methylation data was comparable to that previously reported for Arabidopsis and tomato (Lister et al., 2008; Lang et al., 2017), indicating that our single-base resolution DNA methylation data were sufficient for subsequent analysis.

**Figure 1:**
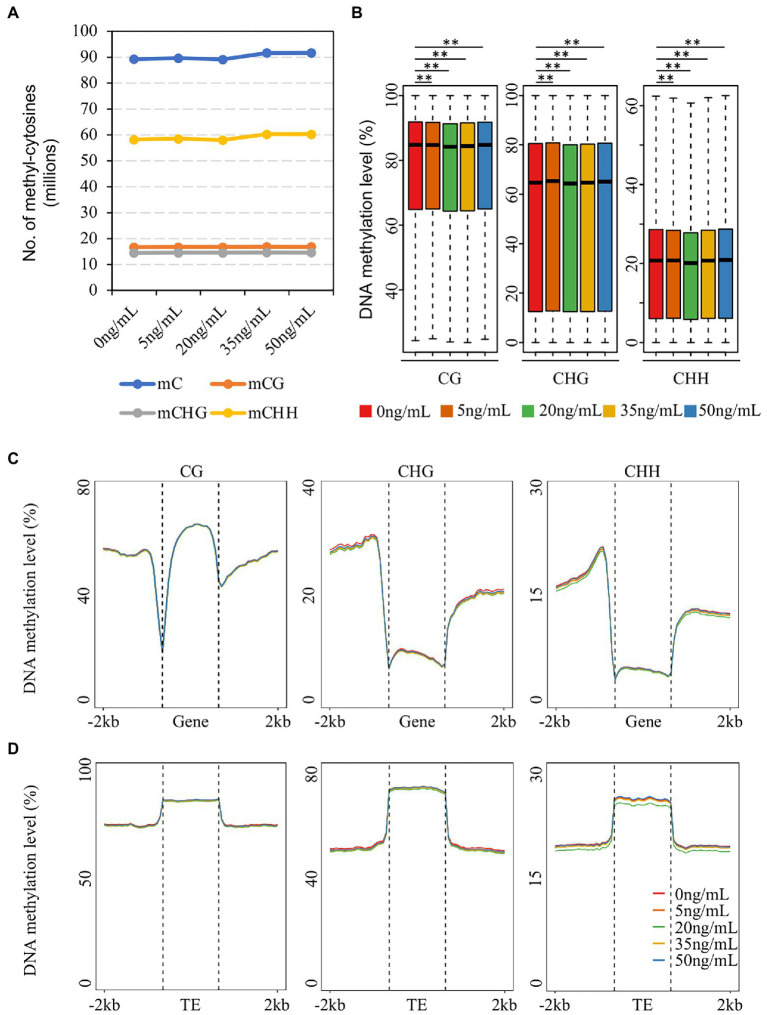
Characteristics and comparison of DNA methylation of potato treated with different DON concentrations. **(A)** The number of methyl-cytosine of CG, CHG and CHH context of each sample. **(B)** Distribution of methylation levels in 200 bp windows after five DON concentration treatments (Mann–Whitney test *p* < 0.001). **(C,D)** Metaplot showing CG, CHG, and CHH methylation levels and patterns of gene **(C)** and TE **(D)** regions after different DON concentration treatments.

The proportion of methylated cytosines varies across plant species. The genome-wide methylated cytosines of potato tubers treated with different concentrations of DON were defined using binomial tests, as described previously (Huang et al., 2019). In potato, ~37% of the total cytosines were methylated after 0 ng/ml DON treatment, which is higher than that in tomato (22%). Among them, cytosines in the CHH (~38%) context were lower than those in the CG (63%) and CHG (48%) contexts. The proportion of methylated cytosines in potato tubers after 35 and 50 ng/ml DON treatments was 1% higher than that after 0 ng/ml. The number of CG- and CHG-methylated cytosines barely changed at different DON concentrations (Figure 1A). In contrast, the number of CHH methylated cytosines showed a slight increase at 35 and 50 ng/ml compared to that at the other treatments. We then investigated the genome-wide average CG, CHG, and CHH methylation levels and found that the DNA methylation levels in potato tubers were very similar after treatment with different concentrations of DON (Supplementary Figure 3), in contrast to rice, which showed a slight increase in DNA methylation levels during *Magnaporthe oryzae* infestation (Cui et al., 2021). We divided the whole genome into 200 bp regions and calculated the average methylation levels of CG, CHG, and CHH for each region. The results showed that the methylation levels were mainly distributed between 60% and 90%; and the CG methylation levels in these regions were notably higher than those in CHG and CHH. Although there was no significant difference in the distribution of the average methylation levels of the three sequence contexts for all 200 bp regions after DON treatment, the methylation levels of the four concentrations (5, 20, 35, and 50 ng/ml) showed significant variation compared with the control (0 ng/ml; Mann–Whitney test, *p* < 0.001, Figure 1B), suggesting that treatment of potato tubers with DON altered the DNA methylation levels across the entire genome.

To investigate the patterns of methylation in different genomic structures, we analyzed the DNA methylation profiles of genes and TE regions and found no significant changes in the three contexts of genes and flanking regions after DON treatment, which was consistent with the global pattern of DNA methylation (Figure 1C). However, CHH methylation in the TE and flanking regions was significantly lower with DON treatment at 20 ng/ml compared to that at the other treatments (Figure 1D). In angiosperms, LTR-RTs (Copia and Gypsy) constitute the largest proportion of genomes, and different types of transposons are regulated by various DNA methylation pathways (Ji et al., 2019; Mhiri et al., 2022). We investigated the changes in CHH methylation in three types of TEs and flanking regions after treatment with five concentrations of DON (Supplementary Figure 4) and found that the methylation patterns of LTR-RTs (Copia and Gypsy) were consistent with all TEs, suggesting that the changes in CHH DNA methylation in potato tubers after treatment with 20 ng/ml DON occurred mainly in the LTR-RT regions. In summary, although different concentrations of DON treatment caused slight changes in genome-wide methylation levels in potato tubers, these methylation variations occurred mainly in the transposon regions, especially the LTR-type TEs.

### Association Between DNA Methylation and Gene Expression

**Figure 2:**
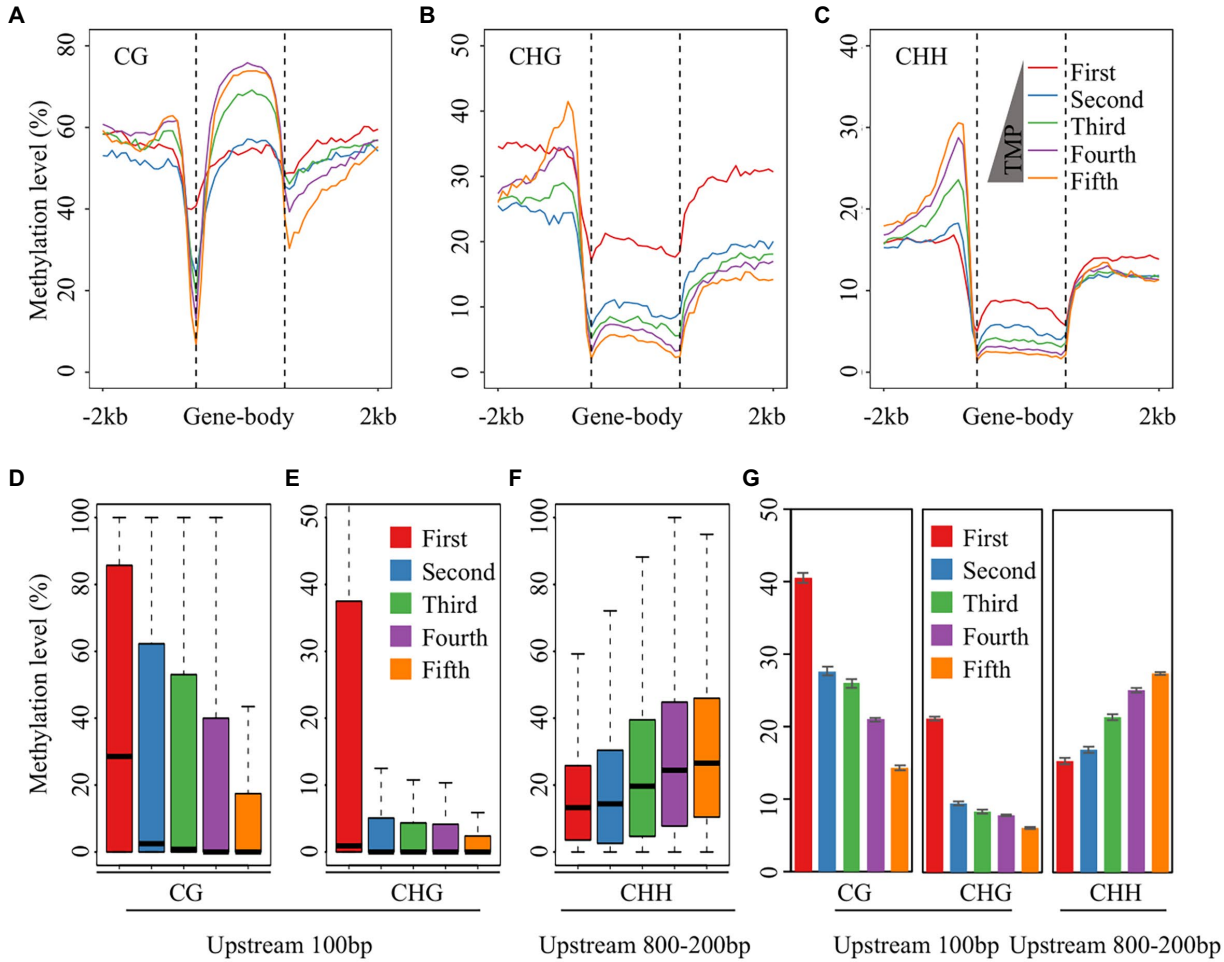
Correlation analysis between gene expression and DNA methylation. **(A–C)** Comparative analysis between gene expression and DNA methylation for the CG **(A)**, CHG **(B)**, and CHH **(C)** sequence contexts. The expressed genes were equally divided in to four groups according to the expression value from low to high, except for the first group of genes whose expression value was 0. **(D,E)** The distribution of CG **(D)** and CHG **(E)** methylation levels of the 100 bp upstream of TSS. **(F)** The distribution of CHH methylation levels of 200–800 bp upstream of TSS. **(G)** The average CG and CHG methylation levels of the 100 bp upstream of TSS, and the average CHH methylation levels of the 200–800 bp upstream of TSS after five DON concentration treatments.

Previous studies have demonstrated that DNA methylation associated with genes occurs in the proximal or gene body regions and regulates gene expression (Jones, 2012; Wang et al., 2015). Generally, DNA methylation in the upstream region is negatively correlated with gene expression. However, some cases showed a positive correlation between the methylation of upstream regions and gene expression levels (Lang et al., 2017; Zhang et al., 2018). In contrast to the role of promoter region methylation in gene expression, the role of gene body methylation remains unclear. In plants, genes with high methylation levels in gene body regions are always expressed at high levels, with moderately expressed genes having higher methylation levels than lowly and highly expressed genes (Jones, 2012; Wang et al., 2015; Shi et al., 2021). To investigate the regulation of potato gene expression by DNA methylation after treatment with different concentrations of DON, we divided all genes into five groups according to their expression levels, from low to high expression, and calculated the average methylation level of each group. The results showed that the CG methylation level in the coding region of the fourth group was higher than that in the other groups, which is consistent with previous studies (Figure 2A). In contrast, non-CG methylation levels in the gene body regions were negatively correlated with expression levels, and the group of genes with the highest expression levels (fifth group) had the lowest non-CG methylation levels, indicating that non-CG methylation in the gene body regions may inhibit gene expression (Figures 2B,C).

Apart from the gene body region, the CG and CHG methylation levels decreased sharply 100 bp upstream of the transcriptional start site (TSS), with the fifth group showing the largest decrease in this region and the first group showing only a small decline, suggesting that the methylation levels in this region were negatively correlated with gene expression in the potato tubers (Figures 2D,E). However, CHH methylation levels rose and then fell 200–800 bp upstream of the TSS in all five groups, and the most drastic changes in CHH methylation levels were observed in the fifth group of genes (Figure 2C). Further analysis revealed that CHH DNA methylation levels in the upstream 200–800 bp region were positively correlated with gene expression levels, suggesting that CHH methylation in the upstream region may also promote gene expression (Figure 2F). In addition, we examined the correlation between DNA methylation and gene expression in the upstream region across different concentrations of DON treatment and found that the correlation was consistent at all five DON concentrations (Figure 2G). Taken together, non-CG DNA methylation in the gene body regions and methylation levels in the upstream region near the TSS were negatively correlated with gene expression. Additionally, CHH methylation was positively correlated with gene expression in the upstream region (200–800 bp) of the TSS, implying that CHH methylation in the upstream region may play a role in promoting gene expression.

### Identification of Differential Methylation Regions After DON Treatment at Different Concentrations

**Figure 3:**
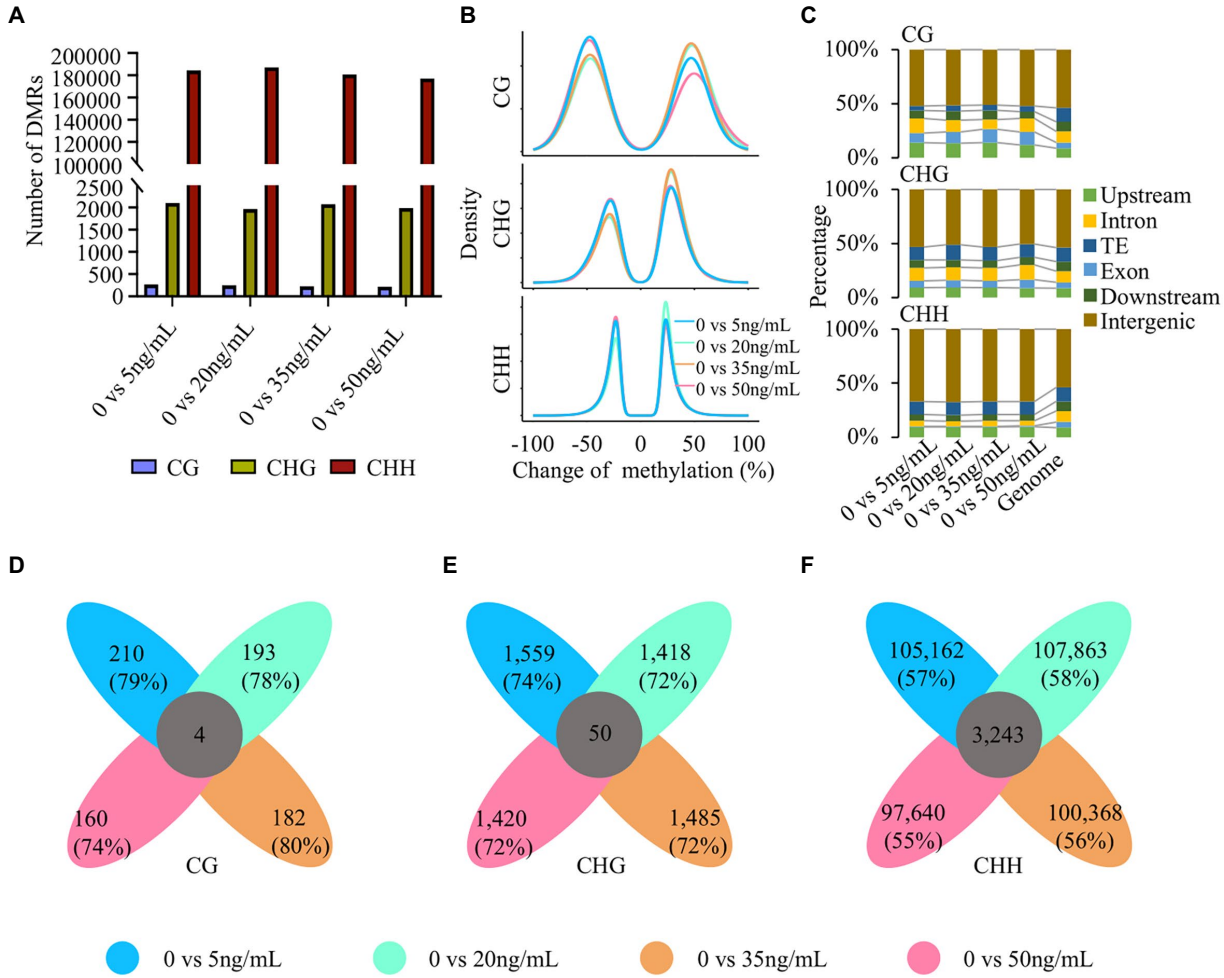
Differential methylation regions (DMRs) after treatment with different DON concentrations. **(A)** The number of DMRs at five DON concentrations. Zero nanogram per milliliter was taken as a reference to assess DNA methylation changes. **(B)** The density of differential methylation regions of CG, CHG, and CHH contexts. **(C)** Overlapped genomic features of DMRs. Genome was divided into upstream, exon, intron, TEs, downstream, and intergenic regions. **(D–F)** Venn diagram shows the number of concentration-specific and common DMRs of three contexts.

The above analysis revealed the dynamic changes in DNA methylation in potato tubers treated with different concentrations of DON. As an unbiased and comprehensive assessment of DNA methylation changes, we identified differential methylation regions (DMRs) in the control (0 ng/ml) and four other concentrations (5, 20, 35, and 50 ng/ml). The number of DMRs was similar among the four different concentrations of DON, of which CHH-DMRs were the most abundant, followed by CHG and CG (Figure 3A; Supplementary Figure 5; Supplementary Table 2). The number of CG-DMRs was less than 300 in the different treatments, indicating that methylation changes in the CG type were very limited. In contrast, the number of CHH-DMRs was at least 177,000, implying that CHH methylation plays a more important role than CG and CHG methylation in potato response to different concentrations of DON. Except for CHG- and CHH-DMRs at 20 ng/ml DON and CHG DMRs at 35 ng/ml DON, 45%–55% of the total DMRs were hypermethylated at all other concentrations and sequence contexts (Figure 3B; Supplementary Figure 6). This result indicates that the proportion of hypermethylated and hypomethylated regions after DON treatment at different concentrations was similar. This is different from the findings for rice during blast infection (Cui et al., 2021).

We further analyzed the distribution of DMRs in the genome. Based on the annotation information of the potato genome (Pham et al., 2020), we examined the overlap between DMRs and different genomic structures, such as 2 kb upstream of the TSS, 2 kb downstream of the TES, exons, introns, transposons, and intergenic regions. We found that the majority of DMRs were enriched in intergenic regions, especially CHH-DMRs, with more than 67% of DMRs located at intergenic regions in the 5 ng/ml treatment (Figure 3C). The proportion of CG-DMRs located in the gene and flanking regions was greater than that of non-CG DMRs, and the proportion of CHH-DMRs distributed in exonic regions was extremely small. Moreover, this phenomenon was consistent across different concentrations of DON.

We found that the characteristics of DMRs were relatively similar across different concentrations of DON. Next, we investigated the specificity of DMRs induced by DON treatment at different concentrations in the potato genome. Unexpectedly, at least 70% of the CG- and CHG-DMRs were concentration-specific, and the concentration-specific CHH-DMRs were > 55% (Figures 3D–F). This finding indicates a high concentration specificity of DNA methylation changes across different concentrations of DON. The percentages of hypomethylation and hypermethylation, as well as the distribution of concentration-specific DMRs, were consistent among all the analyzed DMRs (Supplementary Figure 7). In summary, extensive changes in CHH methylation occurred mainly after DON treatment. The high proportion of concentration-specific DMRs implied that DNA methylation may participate in different biological pathways in response to DON stress.

### DMRs Associated With Transcriptional Changes

**Figure 4:**
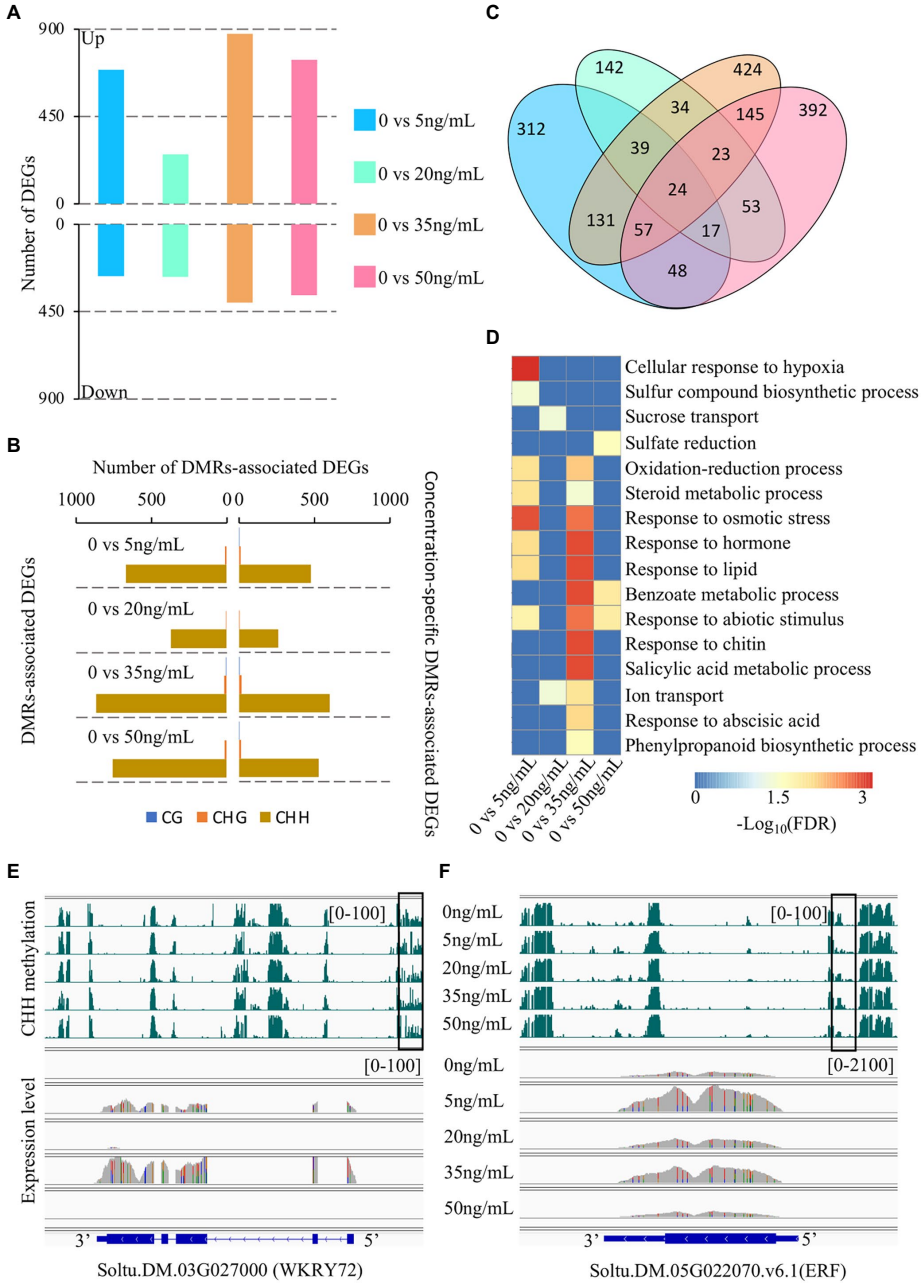
Characterization of differential methylation regions (DMRs)-associated differentially expressed genes (DEGs). **(A)** The number of DEGs after different DON concentration treatments, 0 vs. 5, 20, 35, and 50 ng/ml. **(B)** The number of DMR and concentration-specific DMRs-associated DEGs. **(C)** The Venn diagram shows the number of specific DMRs-associated DEGs. **(D)** Enriched gene ontology (GO) terms for DMR associated DEGs after different DON concentration treatments. **(E,F)** Snapshots of CHH DMR-associated DEGs at different concentrations of DON.

To investigate the transcriptional changes in potato tubers treated with different concentrations of DON, we performed RNA-seq of the same samples as those used for BS-seq. Two biological libraries were sequenced for each sample and at least 40 million clean reads were obtained for each library, with more than 85% of the reads mapped to the reference genome (Supplementary Table 3). To assess the reproducibility of the RNA-seq data, we calculated the Pearson correlation coefficient between two biological replicates for each sample. The results showed that the Pearson correlation coefficient between two biological replicates ranged from 0.9 to 0.95 (Supplementary Figure 8), indicating a high reproducibility of our transcriptome data. We identified thousands of differentially expressed genes (DEGs) between the control group (0 ng/ml) and the other treatments (Figure 4A). We found that most DEGs were identified at 35 ng/ml treatment (1,280), followed by 50 ng/ml (1,109), 5 ng/ml (960), and 20 ng/ml (528). At 20 ng/ml, the number of upregulated and downregulated DEGs was similar (256 vs. 272; Figure 4A; Supplementary Table 4). However, at the other three concentrations (5, 35, and 50 ng/ml), the total number of DEGs was approximately twice that at 20 ng/ml, and the number of upregulated DEGs was more than twice as high as the number of downregulated DEGs, indicating that these three concentrations of DON induced dramatic changes in expression in potato tubers.

The above analysis showed that potato had dynamic changes in gene expression and DNA methylation under different concentrations of DON. To investigate the relationship between DNA methylation changes and DEGs, we identified DMR-associated genes from the overlap of DMRs with gene regions (including the gene body and flanking 2 kb regions). We found that at least 70% of DEGs overlapped with DMRs at each treatment concentration, with the number of CHH-DMR-associated genes accounting for at least 97% of the total number of DMR-associated genes (Figure 4B; Supplementary Figure 9). Further analysis showed that ~70% of the DMR-associated DEGs were concentration-specific DMRs: 5 ng/ml (488/686), 20 ng/ml (265/379), 35 ng/ml (621/894), and 50 ng/ml (542/778; Figure 4B). We also observed a high concentration specificity of DMR-associated DEGs at different concentrations of DON (Figure 4C). These results suggest that CHH methylation plays a key role in regulating the transcriptome dynamics of potato tubers in response to DON treatment. We performed GO (Gene Ontology) enrichment analysis to identify enriched functional categories for DMR-associated DGEs for each DON concentration. DMR-associated DEGs of DON treatment at 5 ng/ml and 35 ng/ml concentrations were mainly involved in plant stress resistance, such as oxidation–reduction, sulfur synthesis, and phenylpropanoid synthesis. Moreover, the DMR-associated DEGs at 35 ng/ml were significantly enriched in salicylic acid and abscisic acid metabolism pathways (Figure 4D; Supplementary Table 5). Accumulating evidence has shown that transcription factors play an important role in the stress response of plants, thus affecting the ability of plants to tolerate stress (Gibbs et al., 2015; Li et al., 2018). Strikingly, we found that many transcription factors were differentially expressed after treatment with DON at 5 and 35 ng/ml relative to that at 0 ng/ml (Supplementary Table 6). Soltu.DM.03G027000, a WRKY-type transcription factor, plays a role in the basal defense of tomato and Arabidopsis (Bhattarai et al., 2010). We found that it was highly expressed under 5 ng/ml and 35 ng/ml concentrations and overlapped with CHH-DMRs at its upstream region (Figure 4E). Another example is Soltu.DM.05G022070, which was highly expressed under 5 ng/ml and 35 ng/ml concentrations and also contained a CHH-DMR at the promoter region (Figure 4F).

Nucleotide-binding sites-leucine-rich repeat (NLR) proteins are the most important resistant proteins that play an important role in plant disease resistance (Meyers et al., 1999). Changes in the expression of these genes are key factors in stress-induced responses of plants. To investigate the functional roles of NLR genes under the different treatment concentrations, we identified 421 NLR genes in the potato genome using a similarity search (Supplementary Table 7). In total, 27 of these genes were DEGs across the different treatments, of which 20 were DMR-associated DEGs (Supplementary Table 8). This finding suggests that DNA methylation is involved in the regulation of NLR gene expression. Collectively, we found numerous DEGs accompanied by altered methylation levels in the 5 ng/ml and 35 ng/ml treatments. In particularly, many of these genes were involved in stress resistance pathways. However, the other concentrations affected the genes involved in stress response, but not as strongly as those in the 5 and 35 ng/ml treatments. The mechanism for this remains unclear, warranting further research.

### Effect of Different Concentrations of DON Treatment on the Area of *Fusarium* Infestation Lesion

**Figure 5:**
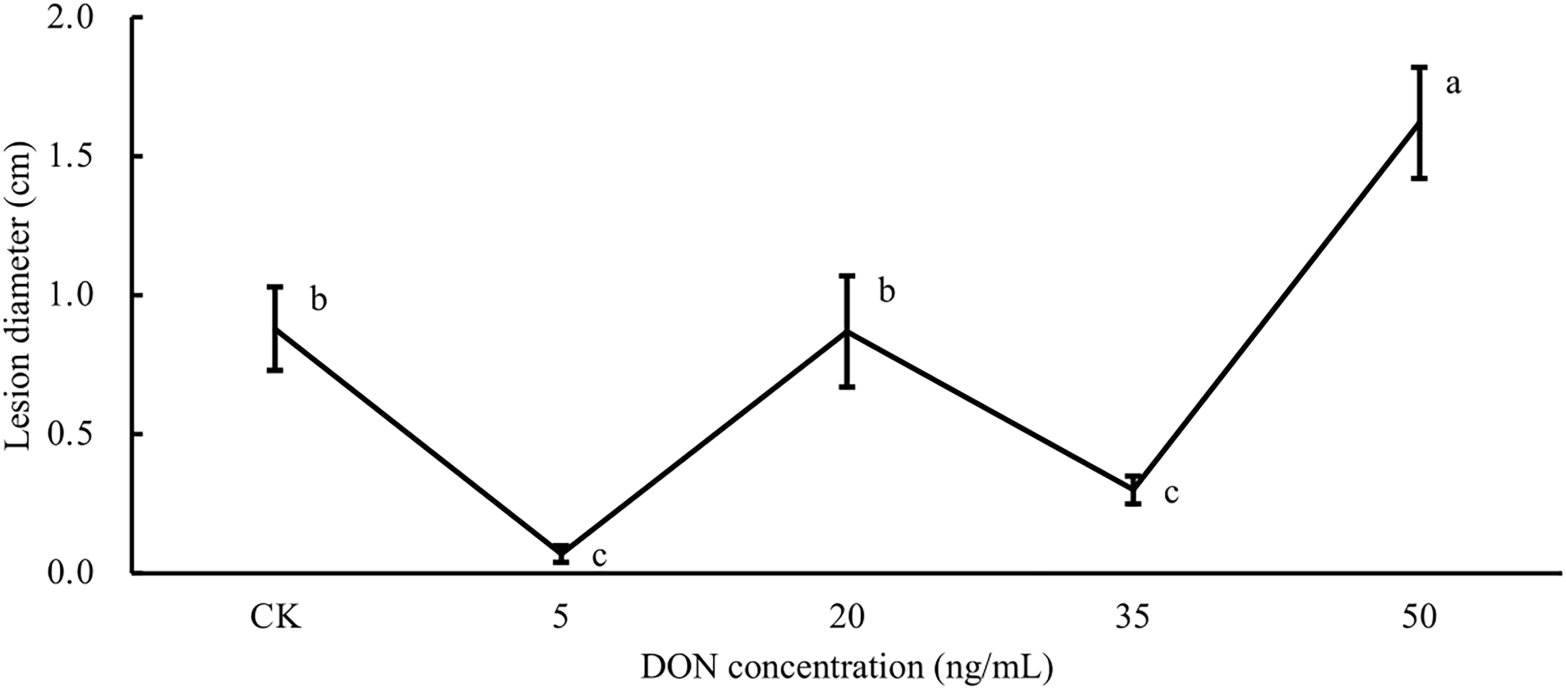
Potatoes treated with different DON concentrations were inoculated with *Fusarium sambucinum*. Comparative analysis of potato tuber lesion diameters after different DON concentration treatments.

It has been shown that DNA methylation changes occurring in response to environmental stress can create stress memory in plants, thus, producing a faster and stronger stress resistance response to the same or similar stresses (Song et al., 2020). From the above analysis, we found that 5 and 35 ng/ml DON treatments may have produced resistance responses in potato tubers, and numerous DMRs were found to be associated with these resistance responses. As DON is a secondary metabolite of *Fusarium* infestation of potato tubers and a virulence factor that aids *Fusarium* infestation, we hypothesized that methylation changes in these regions might create epigenetic memory and establish induced resistance (IR), which enhances the resistance of potato tubers to dry rot. We used *F. sambucinum* to inoculate potato tubers that had been treated with five different concentrations of DON for 6 h. By 10 days after *Fusarium* inoculation, we examined the diameters of lesion on potato tubers. As shown in Figure 5, the lesion area varied greatly under different treatments. Potato tubers treated with 5 ng/ml DON had the smallest lesion diameter, which was 92% lower than that of the control group. Similarly, 35 ng/ml DON treatment also significantly reduced lesion diameter relative to the control (*p* < 0.05), but the lesion diameter was slightly larger than that of the 5 ng/ml treatment. Unexpectedly, when the treatment concentration was 20 ng/ml, the potato tuber lesion diameter was close to that of the control and greater than that of 5 ng/ml and 35 ng/ml DON treatments. At a concentration of 50 ng/ml, the lesion diameter reached 1.5 cm, implying that the whole potato was almost completely rotten. In conclusion, treatment with 5 ng/ml and 35 ng/ml DON significantly enhanced potato resistance to dry rot, probably due to the establishment of IR.

## Discussion

Potato dry rot caused by *Fusarium* is a major threat to potato production and consumption worldwide. *Fusarium* infection is not only associated with rotting tubers but also the accumulation of mycotoxins, raising additional health concerns for consumers. The limited number of resistant varieties and emergence of currently available fungicide-resistant breeds make it an even greater threat to potato cultivation and trade. IR in plants, triggered by the stimulation of certain biotic or abiotic factors, enhances plant resistance and adaptation to environmental changes. DNA methylation plays important roles in the regulation of gene expression (Meng et al., 2016), biotic and abiotic stress responses (Alonso et al., 2019; Eriksson et al., 2020; Kumar et al., 2020), and environmental adaptation (Gáspár et al., 2019; Varotto et al., 2020). In recent years, researchers have suggested that plant immunity is regulated by DNA methylation (Luna et al., 2012). Our laboratory found that DON can induce enhanced resistance to dry rot in potatoes. To explore the regulatory mechanisms of DNA methylation that induce resistance to dry rot, we constructed DNA methylation profiles at a single-base resolution and transcriptome profiles of potato tubers treated with different concentrations of DON. We found slight changes in DNA methylation after DON treatment relative to that in the control. However, we identified thousands of differentially methylated regions across different treatments, suggesting that DNA methylation responds to DON treatment through local variation. Similarly, inorganic phosphorus starvation mainly resulted in CHH hypermethylation in localized regions of the rice genome (Secco et al., 2015), whereas global DNA methylation levels were similar in untreated rice and rice infected with rice blasts (Cui et al., 2021). Additionally, DNA demethylation plays an important role in cold stress tolerance in Arabidopsis (Conde et al., 2017a,b).

Recent studies showed that DON treatments induced the expression of defense genes and the production of reactive oxygen species (ROS) in wheat (Ansari et al., 2007; Desmond et al., 2008; Walter et al., 2008). Meanwhile, DON also induced upregulation of genes involved in the phenylpropanoid and detoxification pathway in barley (Tucker et al., 2021). After treatment with DON at 5 ng/ml and 35 ng/ml, we found that DMR-related DEGs were enriched in plant stress-related pathways, such as phenylpropanoid synthesis and oxidation–reduction, suggesting that DON stress elicited a defense response in potato. We found that 5 ng/ml and 35 ng/ml DON enhanced resistance to dry rot in potato tubers through integrated multi-omics and *Fusarium* infestation experiments, but there were differences in the disease spots after *Fusarium* infestation. We found that DMR-associated DEGs were functionally enriched in hormonal metabolic pathways, such as salicylic acid (SA) metabolism. Choudhury et al. (Heil and Bostock, 2002) described systemic acquired resistance (SAR) and induced systemic resistance (ISR) as two different forms of induced resistance based on the nature of elicitors and regulatory pathways. SAR has been identified as an SA-dependent plant defense characterized by the accumulation of SA and activated expression of pathogenesis-related (PR) genes. Therefore, we hypothesized that these two concentrations of DON induce different forms of IR and that this process is regulated by DNA methylation. In addition, treatment with 50 ng/ml DON reduced resistance to dry rot, implying that high concentrations of DON could promote *Fusarium* infestation in potato. Overall, different concentrations of DON (virulence factor) induced different response pathways in potato, indicating that the dosage of elicitors is a key factor when using elicitors to induce disease resistance in plants.

Plant stress memory involves a variety of physiological, proteomic, transcriptional, and epigenetic changes, and the important role of epigenetic modifications in plant memory has been confirmed in numerous studies (Lämke and Bäurle, 2017). Consequently, we consider that the enhanced resistance of potatoes to *Fusarium* may be due to DNA methylation changes induced by DON. DON at 5 ng/ml induced the strongest resistance and was below the lowest standard of DON limit in cereals set by China (1 mg/kg), which is within the safe level and can be used directly for disease defense. Compared with chemical and biological controls, it has the characteristics of rapid response and low cost. Treatment of Arabidopsis with flagellin, an inducer of plant defense, resulted in epigenetic changes that are stable for at least four generations (Molinier et al., 2006). However, it has been suggested that maintaining epigenetic changes across multiple generations depends on repeated exposure to stress and that these epigenetic changes are stable within only one generation (Boyko et al., 2010). For example, in *A. thaliana*, induced DNA methylation changes can be partially transmitted to the next generation under high salinity stress. However, if the progeny are not continuously exposed to stress, the inherited epigenetic state gradually resets. Thus, this may be a promising new approach for further understanding the mechanisms of epigenetic memory in plant responses to stress.

In conclusion, DNA methylation plays an important role in numerous biological processes, including biotic and abiotic stress responses, and environmental adaptations. In this study, we found that DNA methylation is involved in the regulation of the response of potato tubers to different concentrations of DON stress and may be associated with the establishment of IR in potato. Our results strongly suggest that DNA methylation has great potential for application in plant disease resistance and future breeding studies.

## Data Availability Statement

The data presented in the study are deposited in the NCBI repository, accession number PRJNA832503.

## Author Contributions

HW, FL, and YQ designed the research. YS performed the experiments and analyzed DNA methylation and transcriptome data. YS and HW wrote the paper. All authors contributed to the article and approved the submitted version.

## Funding

This work was supported by fund from National Natural Science Foundation of China grant 32160142 to HW and Science and Technology Innovation Project of Pingtan Science and Technology Research Institute (PT2021007 and PT2021003) to YQ.

## Conflict of Interest

The authors declare that the research was conducted in the absence of any commercial or financial relationships that could be construed as a potential conflict of interest.

## Publisher’s Note

All claims expressed in this article are solely those of the authors and do not necessarily represent those of their affiliated organizations, or those of the publisher, the editors and the reviewers. Any product that may be evaluated in this article, or claim that may be made by its manufacturer, is not guaranteed or endorsed by the publisher.

## Footnotes

^1^ http://ccb.jhu.edu/software/hisat2

^2^ https://ccb.jhu.edu/software/stringtie

^3^ https://github.com/tanghaibao/goatools

^4^ https://www.r-project.org
