# Supplementary material for: Genome-Wide Profiling of DNA Methylome and Transcriptome Reveals Epigenetic Regulation of Potato Response to DON Stress
Source: Front Plant Sci. 2022 Jun 23;13:934379. doi: 10.3389/fpls.2022.934379 (PMC9260311; doi:10.3389/fpls.2022.934379)
Supplement: Supplementary file 1 [file Data_Sheet_1.PDF]

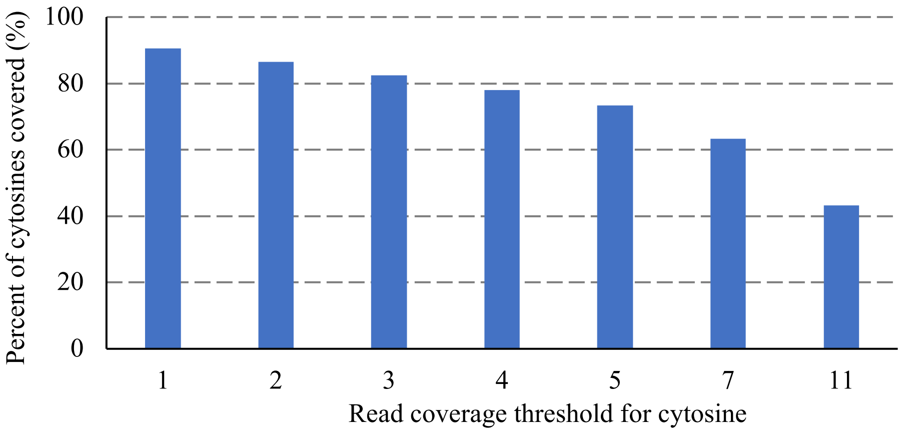

Supplementary Figure 1. BS-seq coverage shown as the proportion of cytosines that were covered by at least X read.

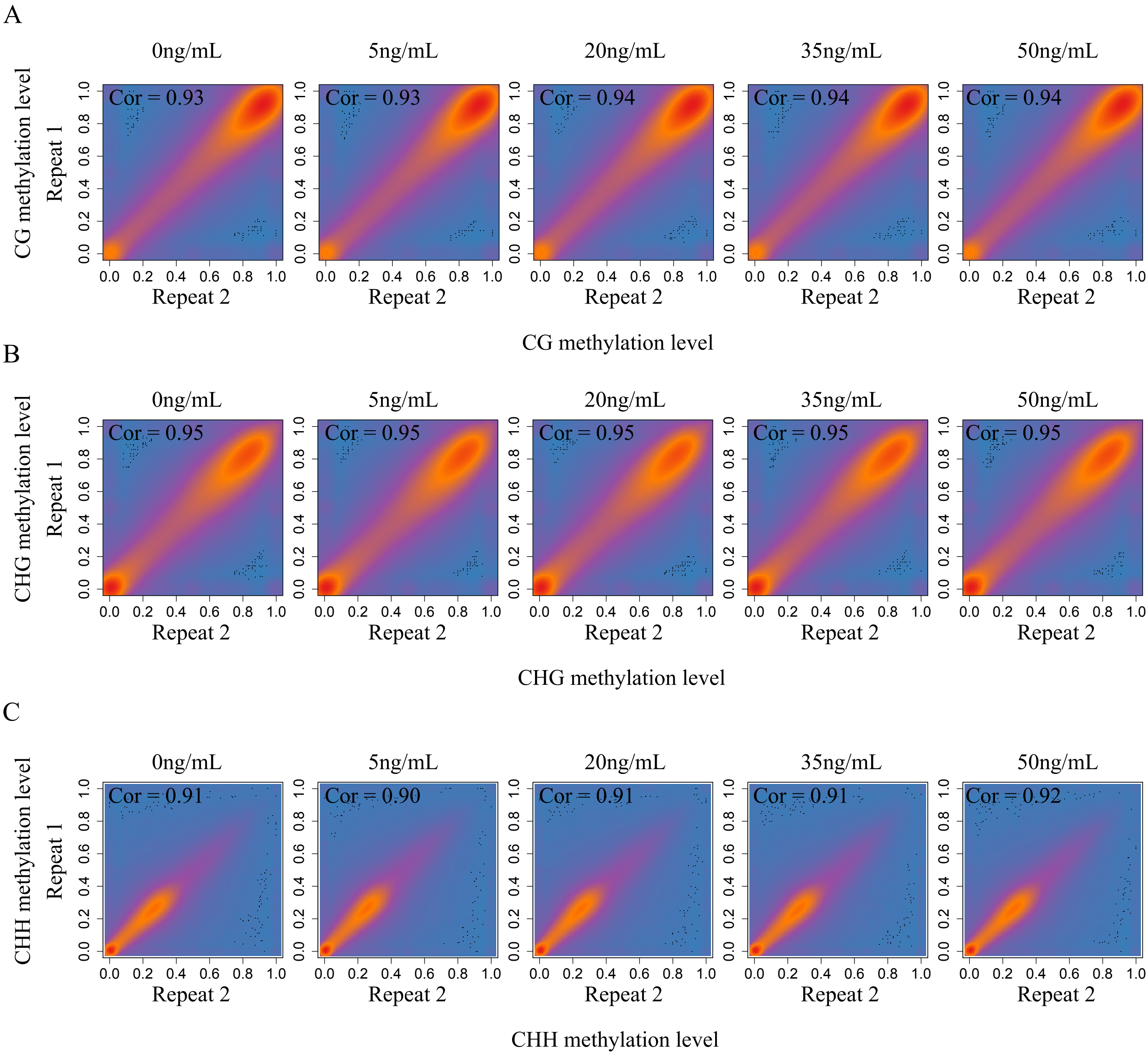

Supplementary figure 2. Evaluation of reproducibility of BS-seq data at five DON concentrations

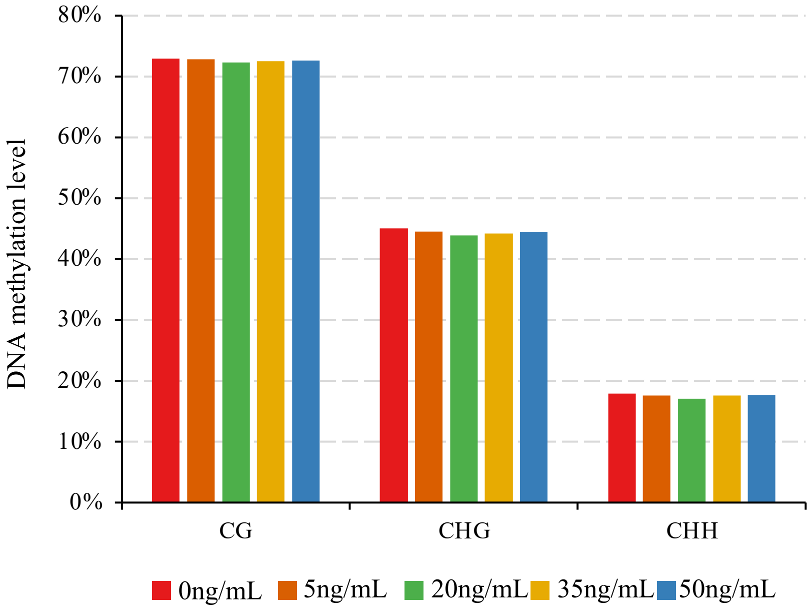

Supplementary Figure 3. Genome-wide weight DNA methylation level after five concentration of DON treatments.

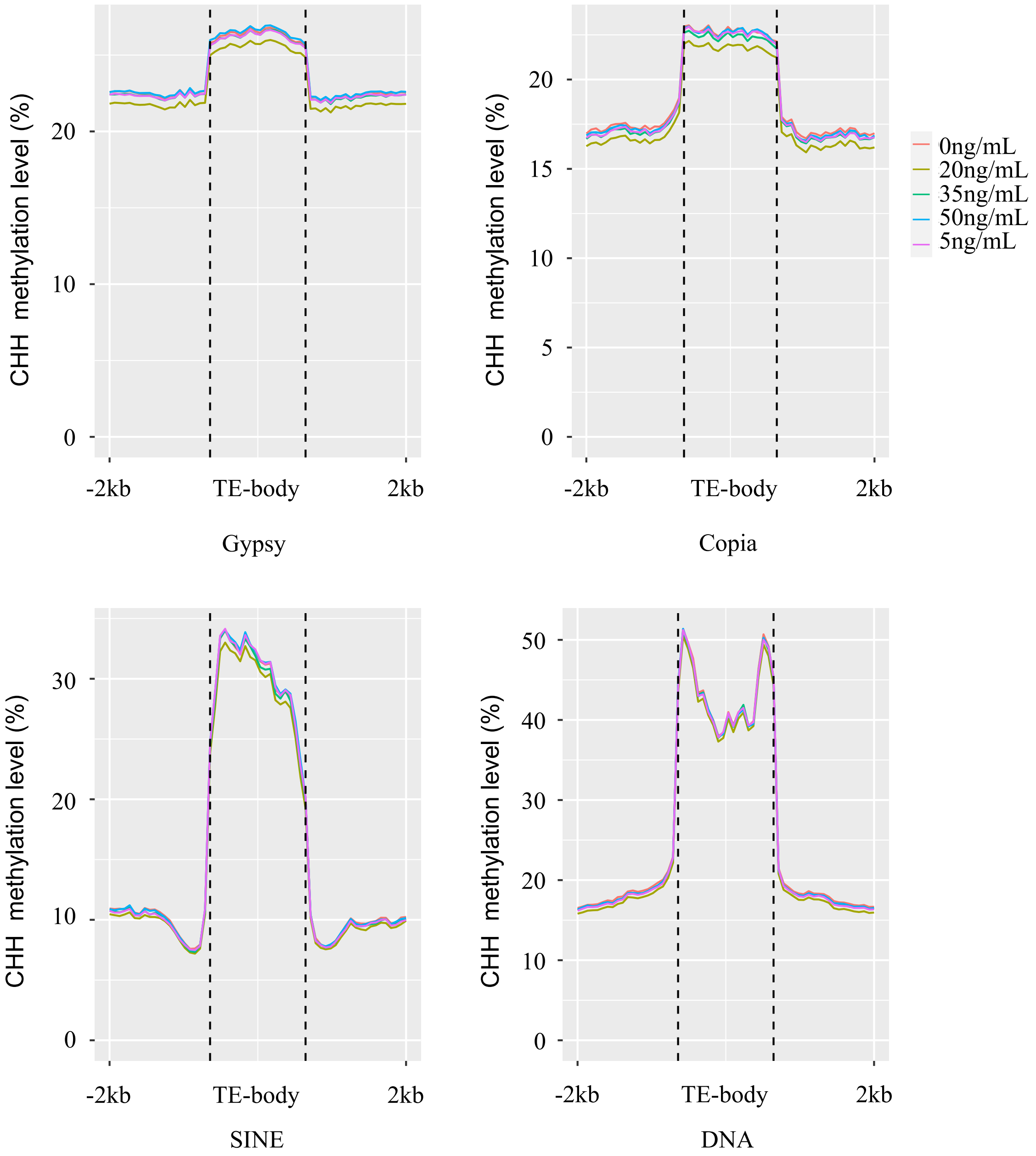

Supplementary Figure 4. Metaplot shows the levels and patterns of CHH methylation in different class of TE regions after treatment with different DON concentrations.

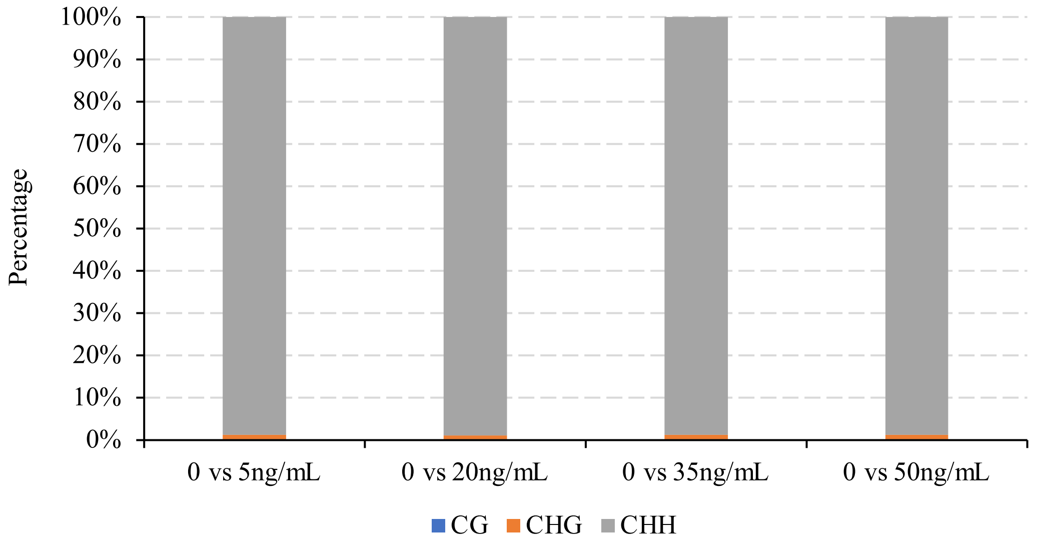

Supplementary Figure 5. Fraction of DMRs at five concentrations of DON.

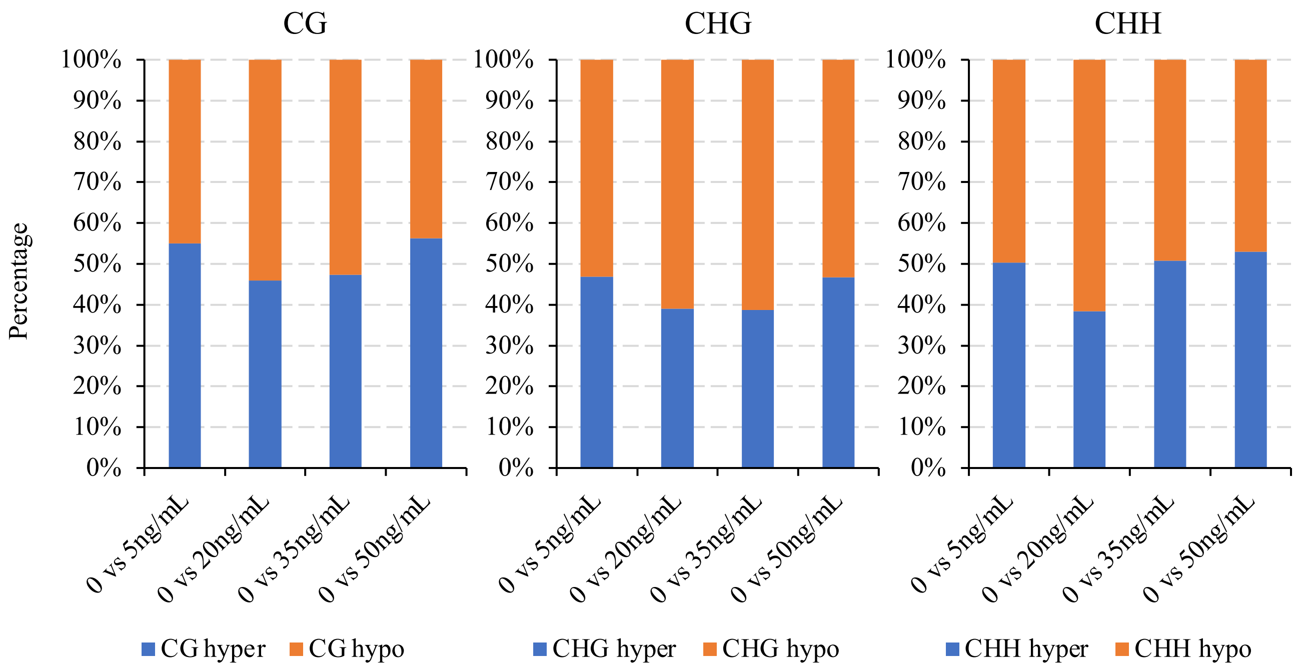

Supplementary Figure 6. Fraction of hyper- and hypo-DMRs after different concentration of DON treatments.

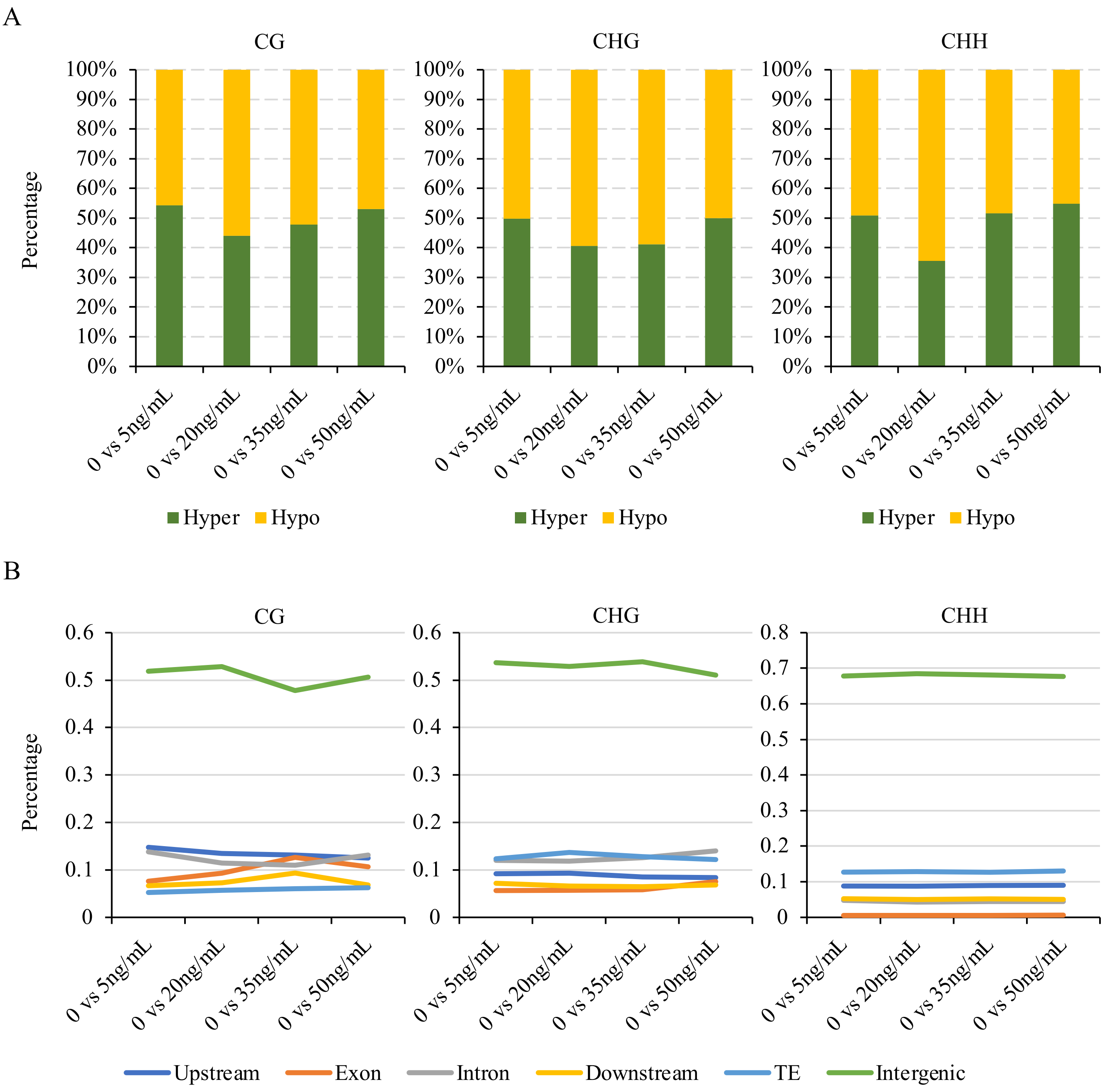

Supplementary Figure 7. Specific concentrations of differentially methylated regions (DMRs) after treatment with different DON concentrations. (A) Fraction of concentration-specific hyper- and hypo-DMRs after different concentration treatments. (B) The genomic distribution of concentration-specific DMRs.

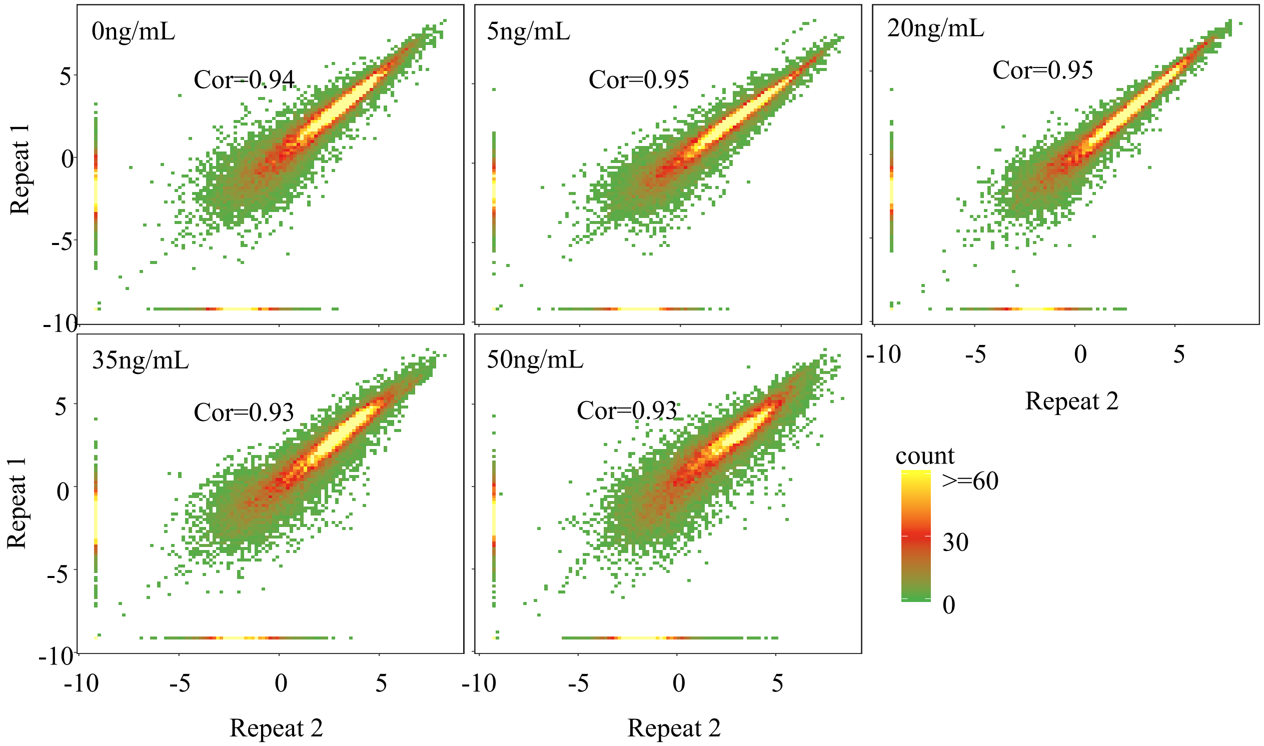

Supplementary Figure 8. Scatter density plot and correlation between two replicates at five concentrations of DON. Cor value indicates the Pearson correlation coefficient between two replicates.

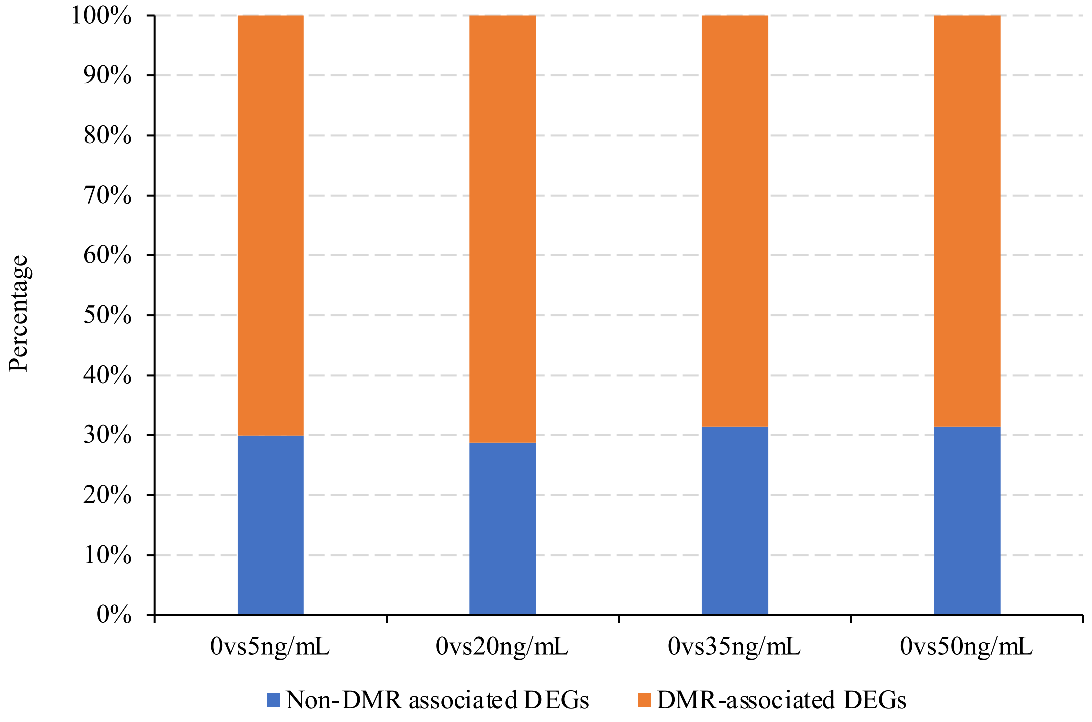

Supplementary Figure 9. Fraction of DMR-associated DMRs after different concentration treatments.
